# Bioclimatic zonation and potential distribution of *Spodoptera frugiperda* (Lepidoptera: Noctuidae) in South Kivu Province, DR Congo

**DOI:** 10.1186/s12898-020-00335-1

**Published:** 2020-11-30

**Authors:** Marcellin C. Cokola, Yannick Mugumaarhahama, Grégoire Noël, Espoir B. Bisimwa, David M. Bugeme, Géant B. Chuma, Adrien B. Ndeko, Frédéric Francis

**Affiliations:** 1 Unit of Crop Sciences, Faculty of Agriculture and Environmental Sciences, Université Evangélique en Afrique, South Kivu, P.O Box: 3323, Bukavu, Democratic Republic of Congo; 2 Functional and Evolutionary Entomology, Gembloux Agro-Bio Tech, Liege University, Passage des Déportés 2, 5030 Gembloux, Belgium; 3 Unit of Applied Biostatistics, Faculty of Agriculture and Environmental Sciences, Université Evangélique en Afrique, South Kivu, P.O Box: 3323, Bukavu, Democratic Republic of Congo; 4 Unit of Geographic Information System, Faculty of Agriculture and Environmental Sciences, Université Evangélique en Afrique, South Kivu, P.O Box: 3323, Bukavu, Democratic Republic of Congo; 5 Faculty of Agriculture, Université Catholique de Bukavu, South Kivu, P.O Box: 285, Bukavu, Democratic Republic of Congo

**Keywords:** Bioclimatic zone, Potential distribution, *Spodoptera frugiperda*, MaxEnt model, Environmental variables

## Abstract

**Background:**

The fall Armyworm (FAW) *Spodoptera frugiperda* (JE Smith), is currently a devastating pest throughout the world due to its dispersal capacity and voracious feeding behaviour on several crops. A MaxEnt species distributions model (SDM) was developed based on collected FAW occurrence and environmental data’s. Bioclimatic zones were identified and the potential distribution of FAW in South Kivu, eastern DR Congo, was predicted.

**Results:**

Mean annual temperature (bio1), annual rainfall (bio12), temperature seasonality (bio4) and longest dry season duration (llds) mainly affected the FAW potential distribution. The average area under the curve value of the model was 0.827 demonstrating the model efficient accuracy. According to Jackknife test of variable importance, the annual rainfall was found to correspond to the highest gain when used in isolation. FAWs’ suitable areas where this pest is likely to be present in South Kivu province are divided into two corridors. The Eastern corridor covering the Eastern areas of Kalehe, Kabare, Walungu, Uvira and Fizi territories and the Western corridor covering the Western areas of Kalehe, Kabare, Walungu and Mwenga.

**Conclusions:**

This research provides important information on the distribution of FAW and bioclimatic zones in South Kivu. Given the rapid spread of the insect and the climatic variability observed in the region that favor its development and dispersal, it would be planned in the future to develop a monitoring system and effective management strategies to limit it spread and crop damage.

## Background

The Fall Armyworm (FAW) *Spodoptera frugiperda* (J.E Smith 1797) is native to tropical and subtropical Americas [14, 20] and a major corn pest [30]. Its presence was first reported on the African continent in 2016 [20] and in Asia later on in 2018 [51, 52]. Whether FAW larvae is able to infest more than 80 crop species [18, 46], main damages were observed on grasses family (Poaceae) including corn, rice and sorghum [34]. Yield losses can reach up to 73% when 100% of the plants are infested with FAW [27]. According to Baudron et al. [5], maize infestation of 54.9% might have an impact on yield of approximately 12%. Due to its polyphagous feeding behavior and recent introduction in the African continent, FAW is expected to constitute a lasting threat to several important crops in African [20]. Studies on the behavioral characteristics of FAW strains in the Western Hemisphere indicated that two main strains, namely on rice and on maize, are able to mate with each other despite the existence of hybridization barriers [35, 38, 47, 50]. Both rice and maize strains can be found and collected from a single host plant species [29, 37, 47]. Given these characteristics, Nagoshi et al. [36] have even reported that the African infestation may represent a new hybrid population with potentially uncertain behavioral feeding characteristics to become a serious problem for Africa, including Democratic Republic of Congo (DRC).

The fall armyworm has only invaded areas that have a climate pattern similar to the native distribution, justifying the use of climatic Species Distribution Models (SDMs) for further predictive spreading [14]. In recent years, an increasing number of tools for spatial analysis of species distribution at different spatial scales have emerged [21, 28]. These tools have become increasingly popular in ecology. Particularly, niche distribution models were widely used in many ecological applications [41]. In fact, several methods of SDMs, also known as ecological niche modeling (ENM) have been developed [19]. In order to estimate the functional response related to various environmental variables [2], SDMs relate known locations of a species with their environmental characteristics, and then predict the potential geographical range of that species [17]. According to Westbrook et al. [56], the initiation and displacement patterns of insect migrations are dependent on these environmental factors.

Distribution of FAW has been investigated by Wang et al. [55] and Liu et al. [32] using SDM MaxEnt (Maximum Entropy). Also, the FAW distribution was modeled on a large scale using CLIMEX software integrating the species model “Wet tropical” [13]. Using similar software and two general circulation models (GCMs), Ramirez-Cabral et al. [49] assessed the climate change impact on future suitability for FAW expansion. Furthermore, Early et al. [14] used Species distribution models (SDMs) to forecast FAW global extent. However, FAW occurrence in South Kivu (Eastern DR Congo) has been reported by Cokola [10] but its distribution remains unknown. Several areas in South Kivu are favourable to FAW development according to suitable temperature, day length and precipitation during warm/wet season as provided by Abraham et al. [1].

Modeling potential distribution of species in relation to climatic conditions is an important tool to apply such as in South Kivu where FAW geographical distribution is still unknown. A FAW modeled proposal will be useful for further FAW monitoring and management in case of high scale infestations. Therefore, this study aims to determine bioclimatic zones and establish potential distribution of FAW in South Kivu, eastern Democratic Republic of Congo (DRC).

## Results

### Bioclimatic zones of the South Kivu province

**Fig. 1:**
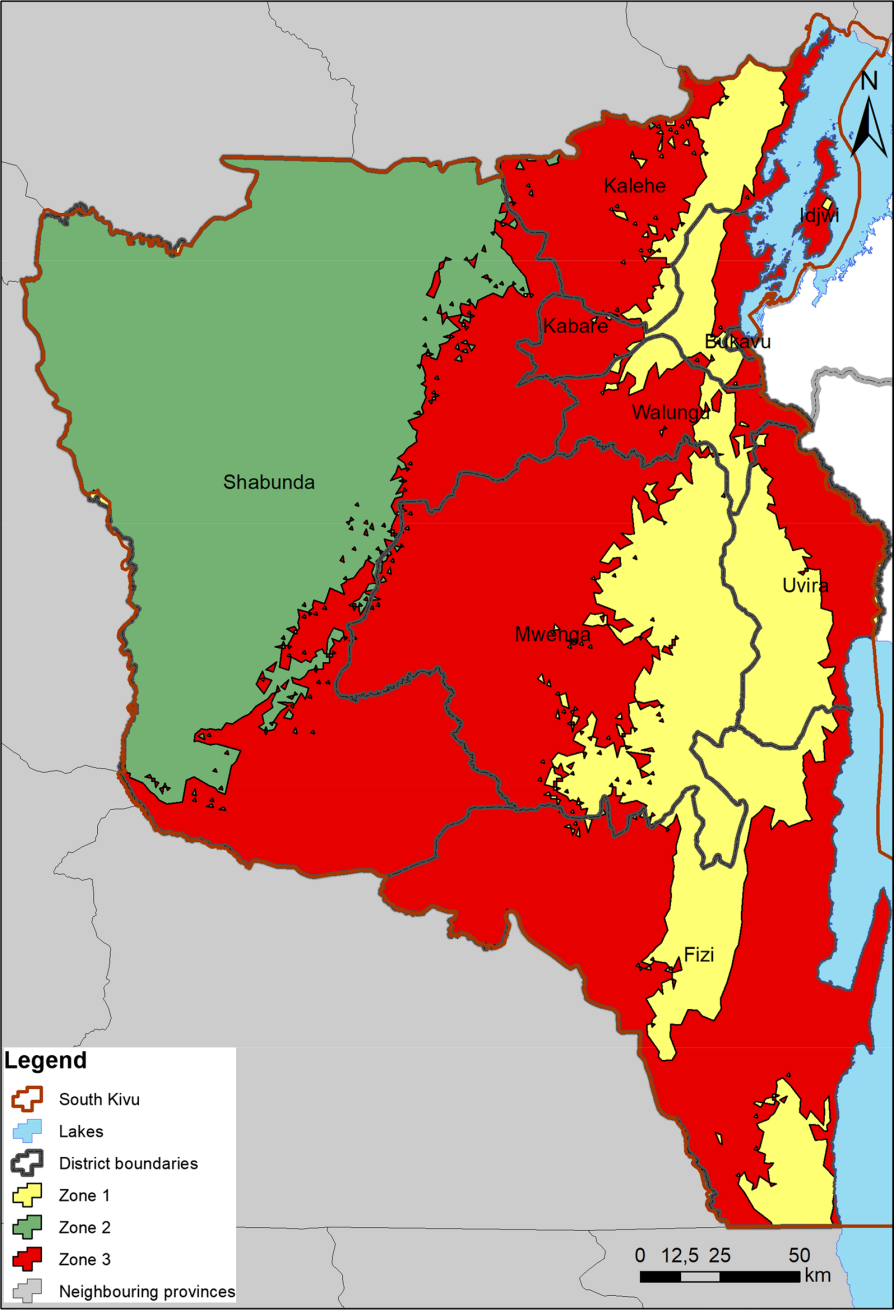
Bioclimatic zones of South Kivu. The zones are indicated in different colors on the map. This figure was created by the authors using ArcMap version 10.6 (https://desktop.arcgis.com/fr/arcmap/)

Three bioclimatic zones obtained by clustering using bioclimatic data were presented (Fig. 1). The respective characteristics (mean ± standard error) of each zone are given in Table 1. Zone 1 is mainly characterized by very high mean daytime temperature range and rainfall parameters (seasonality, duration for the wettest period, in the wettest quarter and annual values). Furthermore, it has very low temperature means (annual, for warmest and coldest quarters, for hottest month and potential evapotranspiration). Also, zone 2 is characterized by very high isothermal and specific rainfall conditions (during driest period, annually, for wettest quarter and moisture index for dry quarter). In addition, it is characterized by very short duration of dry season, very low temperature seasonality and annually, annual moisture index, mean daytime temperature range. Finally, zone 3 was characterized by very high annual temperature and for warmest quarter, longest dry season, very high annual moisture index. However, it was also characterized by very low annual rainfall and for wettest quarter, isothermality and moisture index of the dry quarter. Zones 1, 2 and 3 represented high, low and medium altitude areas respectively.

**Table 1:** Description of bioclimatic zones of South Kivu (Mean ± SE)

Variables	Zone 1	Zone 2	Zone 3	Global
bio1	160.82 ± 19.96	227.28 ± 16.12	220.09 ± 18.06	210.53 ± 30.83
bio2	95.55 ± 4.53	106.89 ± 2.10	105.46 ± 4.75	103.93 ± 5.85
bio3	792.26 ± 39.18	857.40 ± 17.18	790.86 ± 35.53	812.91 ± 44.31
bio4	3.931 ± 0.80	2.99 ± 0.09	3.63 ± 0.80	3.48 ± 0.75
bio5	219.70 ± 23.05	288.97 ± 16.19	285.48 ± 18.98	273.40 ± 33.02
bio6	98.69 ± 16.75	164.26 ± 16.41	152.09 ± 17.09	145.34 ± 29.30
bio7	121.00 ± 9.754	124.70 ± 4.00	133.39 ± 4.15	128.06 ± 7.72
bio10	164.05 ± 20.37	229.63 ± 16.14	224.03 ± 18.06	213.81 ± 30.84
bio11	155.27 ± 20.02	223.59 ± 16.35	215.82 ± 18.31	206.19 ± 31.47
bio12	1893.89 ± 149.49	1940.80 ± 147.15	1563.16 ± 167.94	1753.17 ± 239.61
bio13	248.36 ± 27.76	235.78 ± 24.11	198.67 ± 16.41	220.80 ± 30.48
bio14	17.38 ± 8.03	55.42 ± 13.17	21.59 ± 10.16	31.81 ± 19.79
bio15	80.28 ± 11.02	61.72 ± 6.24	63.03 ± 6.53	66.07 ± 10.41
bio16	668.48 ± 65.06	668.73 ± 62.81	549.53 ± 50.97	612.44 ± 83.07
bio17	89.43 ± 35.89	198.67 ± 34.19	93.86 ± 38.22	127.25 ± 61.75
Dem	2197.31 ± 348.68	847.65 ± 283.62	1145.35 ± 326.32	1259.45 ± 582.61
Llds	2.69 ± 0.81	1.26 ± 1.13	3.29 ± 0.59	2.51 ± 1.23
Mi	147.95 ± 23.30	118.39 ± 8.86	98.43 ± 12.58	114.91 ± 23.68
Miaq	29.30 ± 13.16	50.70 ± 8.95	24.02 ± 9.79	33.81 ± 15.77
Mimq	212.43 ± 35.75	160.83 ± 13.39	140.35 ± 15.53	161.54 ± 34.09
Pet	1295.55 ± 100.38	1640.62 ± 65.71	1595.30 ± 92.60	1549.86 ± 155.42
Total area (km^2^)	11,411.20	17,293.40	30,389.80	59,094.40

*SE* standard error

### Model performance

**Fig. 2:**
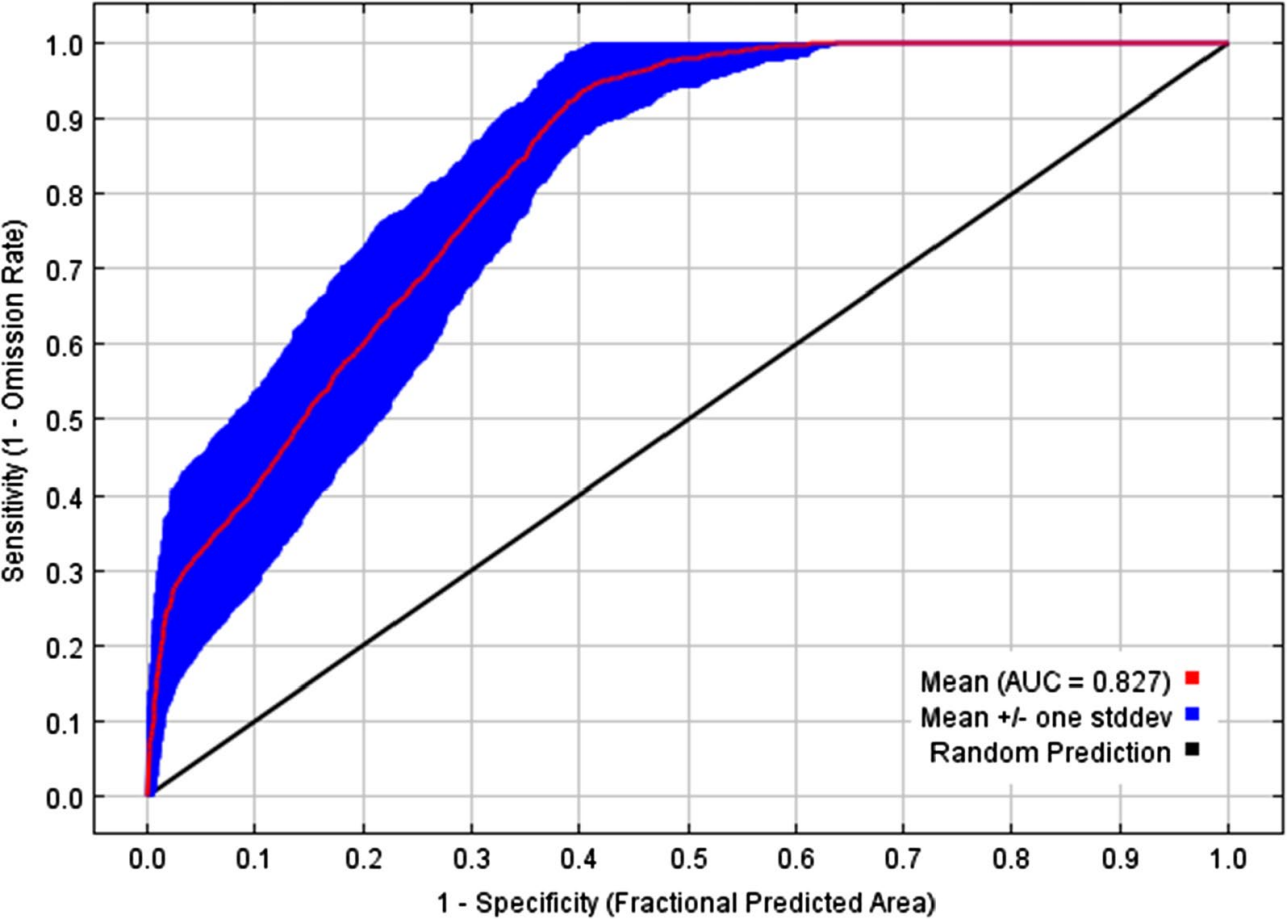
Receiver Operating Characteristic (ROC) curve and Area Under the Curve (AUC) value of MaxEnt modeling (100 runs)

In this study, from the ROC curves, AUC values were used to evaluate the performance of the MaxEnt model. Many studies showed that an AUC of high values leads to better results that significantly differed from the random predictions. The next picture is the receiver operating characteristic (ROC) curve showing the performance of the FAW MaxEnt model. The prediction accuracy of FAW MaxEnt model was found to be acceptable (AUC mean of 0.827 ± 0.033, Fig. 2) according to the identified evaluation criteria.

**Fig. 3:**
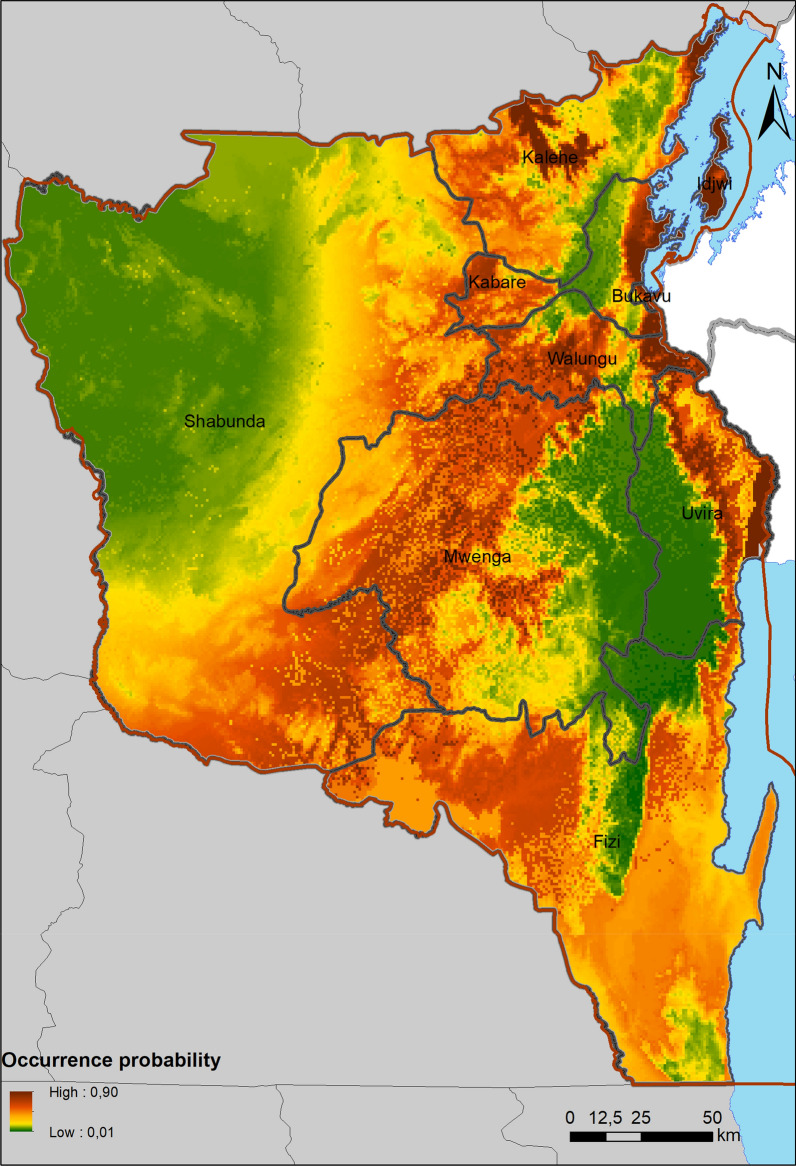
Distribution of suitable areas of fall armyworm (*Spodoptera frugiperda*) in South Kivu, DRC. This figure was created by the authors using ArcMap version 10.6 (https://desktop.arcgis.com/fr/arcmap/)

The suitable areas of FAW in South Kivu province are divided into two corridors (Fig. 3): one covering eastern Kalehe, Kabare, Walungu, Uvira and Fizi territories and another the western areas of Kalehe, Kabare, Walungu and Mwenga territories, southern Shabunda and north-western Fizi territories. The most suitable areas for FAW in South Kivu are mostly located in bioclimatic zone 3. In bioclimatic zones 1 and 2, the probabilities of FAW occurrence are very low (medians below 0.063). As for bioclimatic zone 3, the probabilities of occurrence are relatively higher, with a median of 0.29. In South Kivu, FAW are most likely to be found in areas characterized by very high annual temperature range, longest dry season, very high annual moisture index. Furthermore, these zones are also characterized by very low rainfall (annually, in the wettest quarter, during the wettest month).

### Analysis of variable contributions

**Fig. 4:**
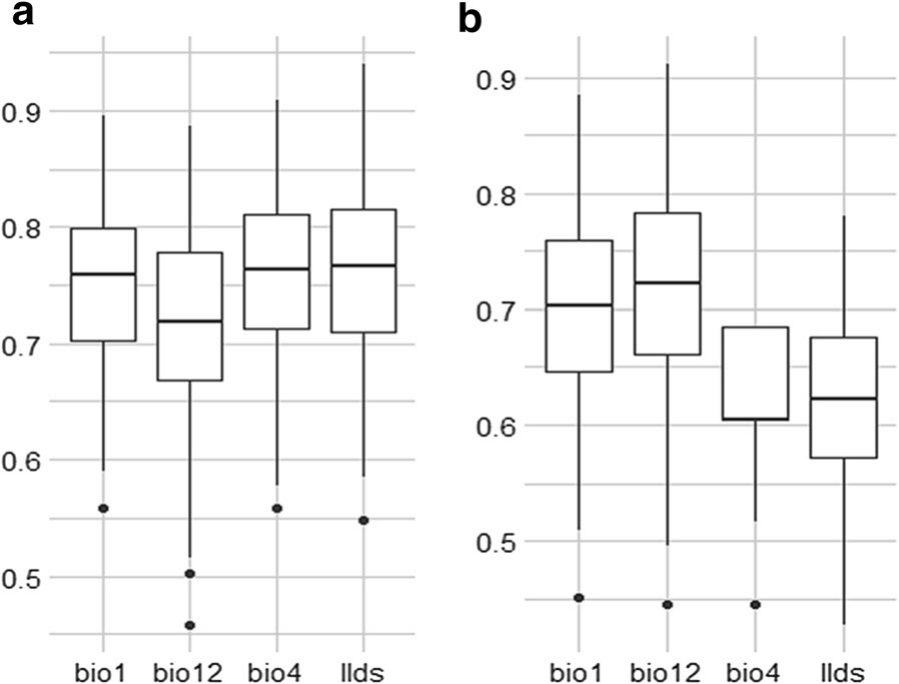
Contribution (**a**) and Permutation importance (**b**) of variables used as predictors in the fall armyworm (*Spodoptera frugiperda*) MaxEnt model. bio1: mean annual temperature; bio12: annual rainfall; bio4: temperature seasonality; llds: longest dry season duration

**Fig. 5:**
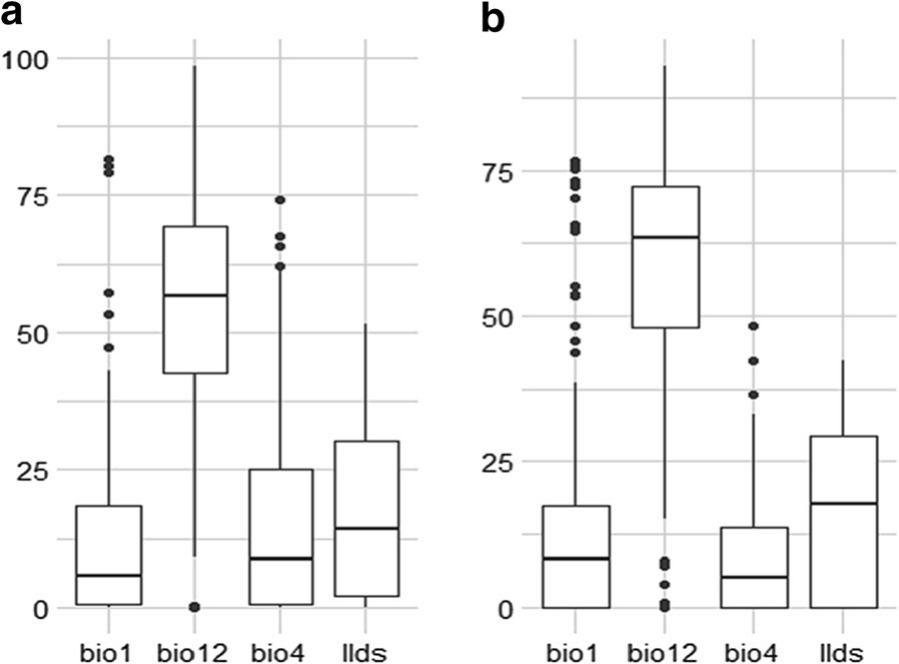
Jackknife test of variables’ contribution in modeling *Spodoptera frugiperda* habitat suitability distribution in South Kivu: **a** without variable, **b** with the variable only. bio1: mean annual temperature; bio12: annual rainfall; bio4: temperature seasonality; llds: longest dry season duration

The estimates of relative contributions of the environmental variables to the FAW MaxEnt model are presented (Figs. 4, 5) showing that bio12 (Annual rainfall) played a major role in the FAW spread. Furthermore, the environmental variable with highest gain when used in isolation was bio12 (Annual rainfall) according to the Jackknife test of variable importance (Fig. 5). This environmental variable also decreases the most the gain while omitted, but also having the most useful information by itself and much more that is not available in the other variables. The bio12 variable was highly correlated with bio7 (Annual temperature range), bio13 (Rainfall of wettest month), bio16 (Rainfall of wettest quarter) and mi (Annual moisture index). Thus, it appears that these four variables also play a major role in the speed of FAW in South Kivu.

### Response of variables to suitability

**Fig. 6:**
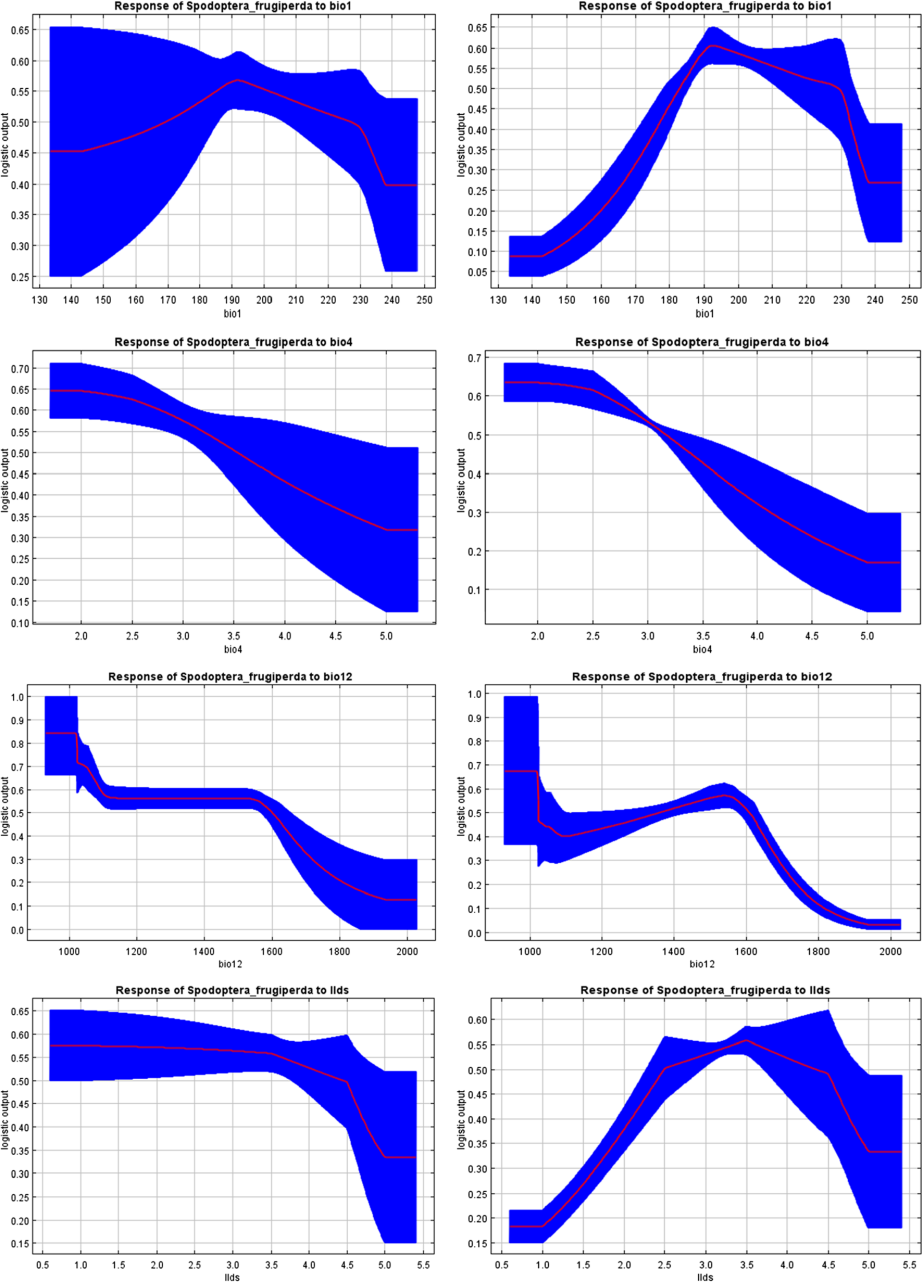
Responses of variables to fall armyworm (*Spodoptera frugiperda*) habitat suitability. These curves show how each environmental variable affects the MaxEnt prediction. They also show how the predicted probability of presence changes as each environmental variable is varied, keeping all other environmental variables at their average sample value (left side) or a MaxEnt model created using only the corresponding variable (right side)

The mean responses of variables to FAW habitat suitability over 100 replicate MaxEnt runs (red) and the mean ± one standard deviation (blue, two shades for categorical variables) are presented. The bio12 (annual rainfall with less than 1600 mm) variable plays a major role in the FAW distribution according to the Jackknife test (Fig. 5). Furthermore, with a strong negative correlation with bio7 (annual temperature range), FAW also favours locations with high annual temperature. The probability of FAW occurrence is high in environments where (1) mean annual temperature (bio1) is comprised between 19 °C and 23 °C; (2) temperature seasonality (bio4) is less than 2.5 and (3) length of the longest dry season (llds) comprised between 2.5 and 4.5 (Fig. 6).

## Discussion

The FAW is a tropical species mostly adapted to warmer parts of the New World [9]. In the current study, we modeled its distribution under tropical conditions in Eastern DR Congo. The existence of 3 bioclimatic zones for FAW was determined in South Kivu. One (zone 3) was found to correspond to the highest probability of FAW occurrence. Climate change has been reported to have different effects on insects, impacting directly their life cycles or indirectly their hosts and/or predators [3, 39]. However, the FAW may benefit from the climate change due to its polyphagous feeding behaviour, its phenotypic and genotypic plasticity [49]. Also, the adult migratory ability is one more adaptative trait to allow moving across regions to several miles (300 miles/generation in some years) [54, 57]. In an area such as South Kivu with an approximate surface area of 69,130 km^2^, the FAW migration would take place very quickly. Outbreaks of FAW are closely related to climate conditions and with good winter and spring conditions [49]. Cokola [10] noted that FAW incidence in South Kivu has been associated by temperature and rainfall. Moreover, study conducted by Liu et al. [32] founded that land-use was more important than climate factors, with larger potential distributions. In this study, among the 21 used bioclimatic variables, four of them influenced the potential distribution of FAW in the region. It is therefore seen that these four variables also play a major role in the spread of FAW in South Kivu. Wang et al. [55] modelled the distribution of FAW through MaxEnt with 19 bioclimatic variables related to temperature and humidity of which 10 influenced the FAW distribution. However, the FAW distribution may be influenced by other several non-climatic factors, such as host, natural enemy, management level and human activities [24], soil properties, land cover and agricultural management interventions (such as use of pesticides or fertilizers) [6]. This aspect need to be then incorporated into the model. Furthermore, it would also be important to model the FAW distribution by integrating local bioclimatic data into the model to minimize errors related to imported bioclimatic data. Soria-Auza et al. [53] reported that one of the least studied sources of uncertainty in species distribution modeling comes from the environmental data used to run the models, particularly the climate data, especially in the tropics, where comparatively few climatic stations are available. In the case of South Kivu province, however, it is difficult to obtain sufficient local bioclimatic data given the limited number of meteorological stations found in this region.

The accuracy of prediction of FAW MaxEnt model showed high values of AUC (Fig. 2) confirming a good model performance [33]. Comparing our results with other studies, including Wang et al. [55], an excellent AUC was found. For instance, AUC often increases with the size of the study area because it contributes to include background points that have environmental characteristics greatly distant from the species requirement, resulting in artificial increase of SDM validation [4]. The suitable areas of FAW in South Kivu province are divided into two corridors (Fig. 3). The Eastern corridor covering the Eastern areas of Kalehe, Kabare, Walungu, Uvira and Fizi territories and the Western corridor covering the Western areas of Kalehe, Kabare, Walungu and Mwenga territories, southern Shabunda and north-western Fizi territories. Infestations are most prevalent in the first corridor. Differences in the FAW infestations within the said corridor, between the Ruzizi plain (low altitude) and Kabare (mid altitude) have been demonstrated [10]. According to the modeling realized by Early et al. [14], Sub-Saharan Africa, especially DR Congo, Gabon and Cameroon, appeared to have low suitability for FAW. Early et al. [14] explain that low suitability in these countries was more likely because of extensive forest cover. This is the case for example, here for Shabunda territory. However, this does not mean that pockets of the suitable habitats in the cited countries will not be severely affected, given the ability of the FAW to travel long distances [14].

Among the four environmental variables used as predictors in the FAW MaxEnt model, bio12 (annual rainfall) played a major role in the spread of FAW and contributed more to run the MaxEnt model (Fig. 5). With the Jackknife test for variable importance, the environmental variable exhibited highest gain when used in isolation with bio12 (annual rainfall). Day et al. [12] found that rainfall in the wettest periods and the coldest annual temperatures were important variables in FAW migration. The effects of rainfall on the distribution of FAW have been documented. For example, Early et al. [14] reported that rainfall have a negative impact on FAW larvae. Furthermore, a suitability map provided by Du Plessis et al. [13] demonstrated that natural rainfall and irrigation scenario were important variables in FAW distribution. The coldest annual temperature and the rainfall during the wettest three months were consistently identified by Early et al. [14] as the environmental variables that most affected FAW distribution. In this work, most suitable habitat for FAW was found in places where annual rainfall was less than 1600 mm. According to Early et al. [14] and Nagoshi et al. [15], FAW was most commonly found in areas with very little forest cover, a minimum annual temperature of 18–26 °C and with 500–700 mm rainfall in the three wettest months. Furthermore, given that variable bio12 is strongly negatively correlated with bio7 (annual temperature range), it seems clear that FAW also favours locations with high annual temperature. Temperature was the main environmental factor affecting the growth and reproduction of the FAW [8, 25]. FAW was most likely to be found in areas characterized by very high annual temperature range, very long duration of the longest dry season, very high annual moisture index, high maximum temperature of the hottest month and very high mean temperature of the warmest quarter. The probability of FAW occurrence is high in environments where mean annual temperature (bio1) is comprised between 19 °C and 23 °C. Du Plessis et al. [22] found that the development rate of FAW increased linearly with increasing temperatures between 18 and 30 °C. Additionally, Wang et al. [55] found that when the Mean Temperature of the Warmest Quarter varies between 19.15 and 29.73 °C, the existence probability of the FAW is higher.

## Conclusion

In areas where investigations on FAW invasions remain limited, such as in the DR Congo, it is important to model its distribution and to detect areas with high infestation potential. Based on the obtained results, the South Kivu province is a favorable habitat for the development of FAW. However, given the rapid spread of the insect and the climatic variability observed in the region that favor its development and dispersal, it is necessary to pay particular attention to the management of this species now, in order to take effective measures and prevent its further spread. At the same time, effective and efficient monitoring systems should be set up in its range to collect field data’s and improve further control of this pest.

## Methods

### Study area and occurrence data collection

**Fig. 7:**
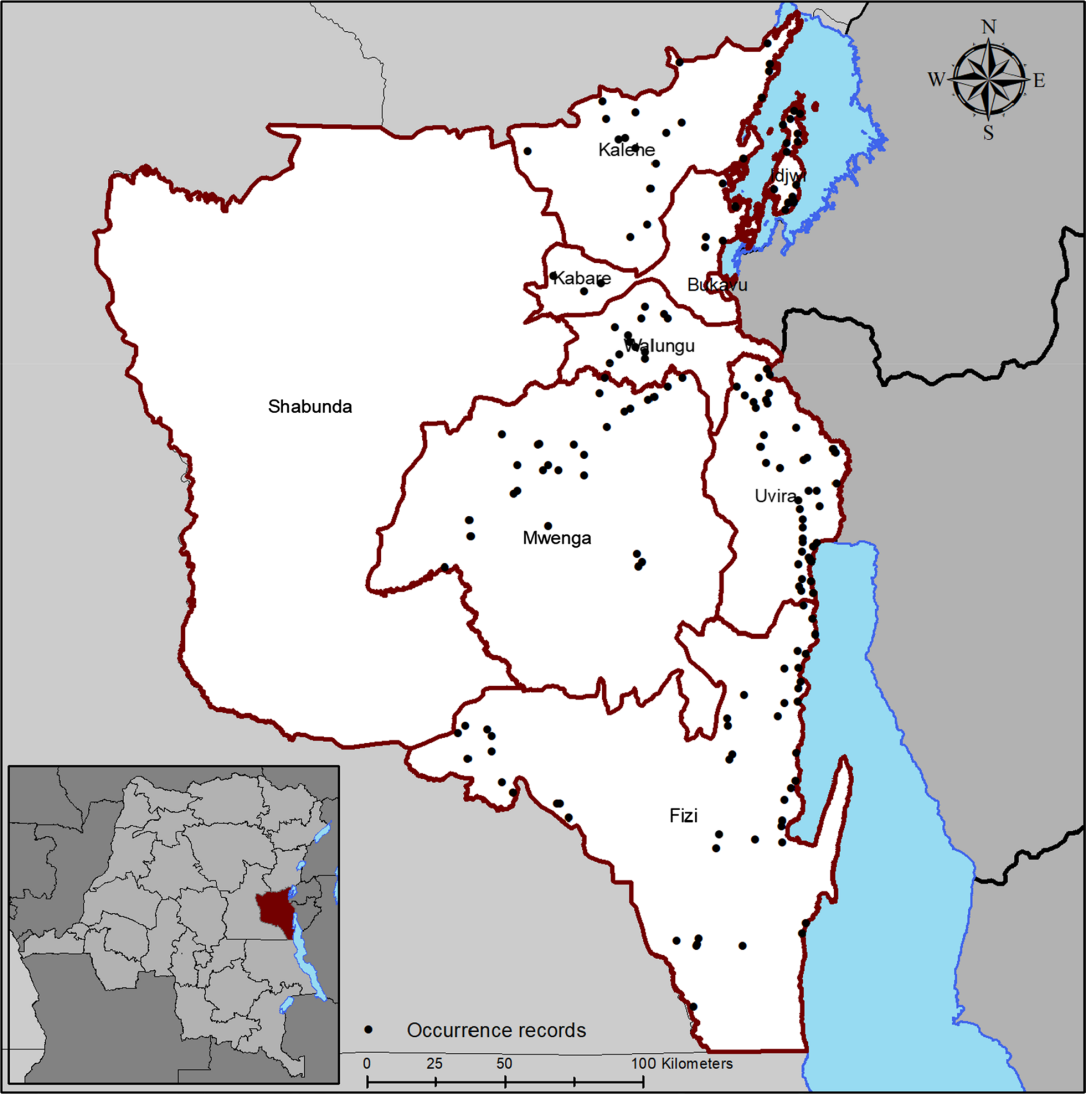
Occurrence records of fall armyworm (*Spodoptera frugiperda*) in South Kivu, DRC. Each point represents a maize field in which fall armyworm larvae were detected and collected. This figure was created by the authors using ArcMap version 10.6 (https://desktop.arcgis.com/fr/arcmap/)

This study focused on South Kivu in Eastern DR Congo, between 1º36′ and 5º South Latitude; 26º47′ and 29º20′ East Longitude*.* Biological data’s related to FAW occurrence were associated to locations with geo-referenced coordinates. Occurrence data of FAW were collected in Kalehe, Kabare, Walungu, Uvira, Fizi, Mwenga and Idjwi territories in collaboration with local farmers who observed FAW larvae and reported every related field in their localities. All suspected cases of FAW attacks were checked for confirmation through field surveys. To confirm that the larvae observed were indeed those of FAW, we had considered the morphological characteristics of FAW larvae as described by EPPO [16] and Sharanabasappa et al. [26]. Geographic coordinates of infested areas were selected only after positive FAW confirmation. Presence records were collected between February 2018 and September 2019 in 156 fields where FAW has been reported. Geographic coordinates on latitude and longitude in the WGS84 system were recorded using GPS Garmin 64 s. The map representing the points of occurrence is illustrated in Fig. 7.

### Environmental variables

In this study, we used elevation and potential evapotranspiration data’s combined with 19 bioclimatic variables. Altitude (Digital Elevation Model ASTERDEM) with 30 m spatial resolution was obtained from USGS database (https://earthexplorer.usgs.gov) and the bioclimatic data’s were collected from the Africlim database (https://www.york.ac.uk/environment/research/kite/resources/). They were used to build the species distribution model in order to find the FAW suitable areas. Africlim provides high-resolution climate data’s for Africa. Bioclimatic data consisted of 21 environmental variables (Table 2) that were obtained from interpolations of monthly averages of precipitation and temperature taking into account climate data collected over long periods of time (1950—2000) [23]. The Africlim spatial database includes monthly grids of temperature and rainfall, deriving from bioclimatic summary variables such as moisture indices and dry season length. All environmental variables were in raster format with a 30 arc seconds resolution (0.93 km × 0.93 km ≈ 0.86 km^2^ at the equator). Both ArcGIS Desktop 10.6 and QGIS 3.10 were used to process the spatial data: data extraction to the South Kivu province extent, data management in geographic coordinates (datum: WGS84) and resampling all the raster layers to the same resolution for preparing the maps.

**Table 2:** Environmental variables used to model *Spodoptera frugiperda* (FAW) distribution in South Kivu

Environmental and bioclimatic parameters	Code	Units
Mean annual temperature (* 10)	bio1	°C
Mean daytime temperature range (monthly average) (* 10)	bio2	°C
Isothermality (bio1/bio7) * 100	bio3	–
Temperature seasonality (standard deviation * 100)	bio4	°C
Maximum temperature of the hottest month (* 10)	bio5	°C
Minimum temperature of the coolest month (* 10)	bio6	°C
Annual temperature range (bio5-bio6) (* 10)	bio7	°C
Mean temperature of the warmest quarter (* 10)	bio10	°C
Mean temperature of the coldest quarter (* 10)	bio11	°C
Annual rainfall	bio12	mm
Rainfall during the wettest month	bio13	mm
Rainfall during the driest month	bio14	mm
Rainfall seasonality	bio15	mm
Rainfall in the wettest quarter	bio16	mm
Rainfall in the driest quarter	bio17	mm
Longest dry season duration	llds	–
Annual moisture index	mi	–
Moisture index of the dry quarter	miaq	–
Moisture index of the wet quarter	mimq	–
Potential evapotranspiration	pet	mm
Elevation	dem	M

### Bioclimatic zonation

Initially, all the environmental variables (n = 21) were clipped to have only spatial data corresponding to the extent of the South Kivu province. Then, geographic coordinates of the raster pixels centroids were used to extract the values for each variables corresponding to each pixel in order to produce a dataset to be used to delineate the bioclimatic zones. The generated bioclimatic dataset was used by processing the Principal Component Analysis (PCA) procedure of the *FactoMineR* [31] package of the R software version 3.5.3 [48]. Based on Kaiser's criterion, only the first 5 principal components were selected for further analysis. The loadings of pixels centroids on the first 5 principal components were then used to perform a hierarchical ascending clustering through the HCPC (Hierarchical Clustering on Principle Components) procedure of the *FactoMineR* package. Hierarchical clustering was realised using the Euclidean distance as the metric and Ward's aggregation method to determine the optimal number of clusters to be formed. The Kmeans procedure was then used to consolidate the obtained clusters. Clustering results were then imported into QGis 3.10 to produce a bioclimatic zone map of the South Kivu province.

### Selection of environmental predictors

Prior to distribution modeling, all the environmental variables were subjected to a correlation test in order to select those susceptible to be used as predictors of the FAW distribution. Consequently, only variables with pairwise Pearson correlation coefficients falling under the interval of ]-0.75, 0.75[ were selected for modeling in order to control for multicolinearity problem in environmental predicators [58].

### Species distribution modeling

MaxEnt (Maximum Entropy) program 3.3.3 [43, 44] was used to establish current climate envelope for FAW natural occurrence in South Kivu. MaxEnt is a common species distribution modeling (SDM) tool used for predicting the distribution of a species from a set of records and environmental predictors [19]. The MaxEnt technique uses known occurrence locations (presence only data) and a set of gridded environmental layers to produce an output map of the predicted ecological niche of the species on a scale of 0 (lowest suitability) to 1 (highest suitability). MaxEnt is a modeling technique that measures entropy, a measure of ‘how much choice’ is involved in the selection of an event [44, 45]. MaxEnt is a general-purpose method for characterizing probability distributions from incomplete information. In estimating the probability distribution defining a species distribution across a study area, MaxEnt formalizes the principle that the estimated distribution must agree with everything that is known (or inferred from the environmental conditions where the species has been observed) but should avoid making any assumptions that are not supported by the data [44]. The approach corresponded to find the probability distribution of maximum entropy (a distribution that is most spread-out, or closest to uniform) subject to constraints imposed by the information available regarding the species observed distribution and related environmental conditions across the study area [44]. MaxEnt was presented as one of the highest performing SDM methods [7].

We ran 100 models, each trained to a randomly selected bootstrap process of the occurrence dataset. Prediction map from each model has been generated in order to calculate the mean prediction and standard deviation of each pixel. Model predictions were imported into ArcGis 10.6 to generate maps of the FAW occurrence probability in South Kivu.

### Model evaluation

In this study, the Receiver Operating Characteristic (ROC) curve method was used to assess the model's performance [11, 40, 42]. One of the parameters used to evaluate predictive capacity of a model generated by MaxEnt is the area under the curve (AUC) or under the ROC curve. AUC can then be interpreted as the likelihood that a randomly selected point of presence is located in a raster cell with a higher probability of species occurrence than a randomly generated point [44]. The AUC is an effective threshold-independent index that can evaluate a model's ability to discriminate presence from absence (or background) occurrence. Also, the AUC is not affected by collinearity and spatiotemporal autocorrelation [11]. The closer AUC is to 1, the more predictive is the model. Random distribution has an AUC of 0.5. Overall value of AUC can be considered in evaluating the final model. AUC values of 0.5–0.7 indicate low accuracy, 0.7–0.9 useful applications and > 0.9 high accuracy [33].

### Assessment of variable contribution

The Jackknife procedure was performed on climate variables to determine the major contributors to the prediction model. The model evaluation was completed by an assessment of the contribution of each variable used in the model based on Jackknife test. However, more detailed evaluation can be carried out during construction of the model by analyzing AUC obtained in different Jackknife test scenario. Then, AUC values obtained from a single variable or with the global models (from which a variable had been removed purposively) can be compared. The main goal in such situation is to identify which variable, when added or removed from the model, mainly modify the AUC value. In this study, the jackknife method was used to analyze the effects of environmental variables on model results in order to select dominant factors. Specifically, the process involves 3 independent steps:

1. Calculating the training gain for the model with only one variable. Higher training gain indicates that the variable has high prediction power and contributes greatly to species distribution;
2. Calculating the training gain for the model without a specific variable and analyzing the correlation between the removed variable and the omission error. If the removal of an environmental variable leads to a significant increase in the omission error, it indicates that the variable has a significant effect on the model's prediction;
3. Calculating the training gain for the model with all variables.

## Data Availability

The datasets used and/or analyzed during the current study are available from the corresponding author on reasonable request.

## Abbreviations

AUC: Area under the curve
ROC: Receiver operating characteristic
MaxEnt: Maximum entropy
SDM: Species distribution model
HCPC: Hierarchical clustering on principle components
PCA: Principal component analysis
CLIMEX: Climate and population modeling software
DRC: Democratic Republic of Congo
